# Uncertainty analysis of single-time-point organ dosimetry compared with the multi-time-point method in radioimmunotherapy

**DOI:** 10.1186/s40658-026-00883-3

**Published:** 2026-05-02

**Authors:** Amit Nautiyal, Gemma Lewis, Jonathan I. Gear, Kim Orchard, Matthew J. Guy, Sofia Michopoulou

**Affiliations:** 1 Department of Medical Physics, University Hospital Southampton, Southampton, SO16 6YD UK; 2 Faculty of Medicine, University of Southampton, Southampton, SO17 1BJ UK; 3 Joint Department of Physics, Royal Marsden NHS Foundation Trust and Institute of Cancer Research, London, UK; 4 School of Computer Science, University of Bristol, Bristol, BS8 1TH UK

**Keywords:** Dosimetry, Single time point, Multiple time point, Anti-CD66, Uncertainty analysis, Radioimmunotherapy

## Abstract

**Background:**

A position paper released by the European Association of Nuclear Medicine emphasised the need for multidisciplinary engagement to establish dosimetry-based personalised treatment in Radionuclide therapy (RNT). The uncertainty analysis results often ignored in routine clinical practice should be incorporated into the dose calculations to improve the efficacy and accuracy of treatment. In this study, patients with haematological malignancies undergoing radioimmunotherapy were evaluated. Our study aimed to calculate the uncertainties associated with each parameter of the single time point (STP) dosimetry chain and compare the with multiple time points (MTP) in the bone marrow and liver results.

**Methods:**

28 patients received an intravenous injection of ^111^In-besilesomab (0.17 ± 0.01GBq) for pre-therapeutic dosimetry and were subsequently treated with ^90^Y-besilesomab(2.43 ± 0.53GBq). A dosimetry analysis was performed on bone marrow (BM) and liver with MTP and STP. We investigated the uncertainty in population mean effective half-life, volume, recovery coefficient, counts, measured activity, fitting parameters, time-integrated-activity, S-factors, and absorbed dose (AD) for a group of patients.

**Results:**

The mean absorbed dose per unit administered activity (DpA) to BM was 5.8 ± 1.7 mGy/MBq with MTP and 5.8 ± 1.6 mGy/MBq with STP, and to the liver was 2.9 ± 1.9 mGy/MBq with MTP and 3.1 ± 2.4 mGy/MBq with STP. The mean fractional uncertainty associated with total absorbed dose to BM was 13.18 ± 3.46% with MTP and 18.75 ± 3.22% with STP, and to liver was 5.77 ± 3.13% with MTP and 49.78 ± 25.36% with STP. A moderate positive relationship (R^2^ = 0.7) was noted between post-injection acquisition time and AD uncertainty with STP for BM, whereas a strong positive relationship (R^2^ = 1) was noted for the liver.

**Conclusion:**

The absorbed dose uncertainty in STP was significantly higher compared to the MTP. Incorporating the uncertainty analysis for STP dosimetry parameters in routine clinical practice is strongly recommended. The accuracy in the acquisition time, population-based half-life and fitting function for time activity curve is vital for minimising uncertainty in STP dosimetry, which is less time-consuming and easier to implement in clinical practice than MTP.

## Introduction

The role of radioimmunotherapy (RIT) has increased rapidly in recent decades for the treatment of various cancers [1]. RIT is a targeted treatment that uses the coupling of monoclonal antibodies (mAbs) with radionuclides to target differentiation antigens. In contrast to external beam radiation therapy (EBRT), RIT can target not only primary tumours but also systemically metastasising lesions [2]. It also allows a higher dose delivery to the target tissue than EBRT, as doses to non-target tissues are lower. In the RIT approach, membrane proteins such as the CD66 antigens (CD: cluster of differentiation; i.e.Anti CD66), or Herceptin (HER) family members are considered to be antigens of interest [3]. In RIT, ß-particle emitting radionuclides that can be conjugated to antibodies are ^90^Y,^177^Lu,^188^Re, and ^131^I [4]. Among them, the rare-earth lanthanide ^90^Y possesses ideal physical (ß-max 2.28 MeV; ß-average 0.94 MeV; T_1/2_ 64.1 h; maximum tissue penetration 11 mm) and cytotoxic properties compared with other isotopes and can be conjugated to mAbs using a chelating agent [5]. A disadvantage of ^90^Y is the lack of gamma photons, which makes conventional scintigraphic imaging for assessing the posttherapy distribution of radioactivity difficult. As a result, surrogate gamma-emitting radioisotopes (e.g., ^111^In-labeled antibodies) were developed and used for ^90^Y dosimetric assessment and pharmacokinetic studies [6].

RIT with anti-CD66 can be used for bone marrow conditioning before stem cell transplantation in the treatment of patients with Myelodysplastic syndromes (MDS) or acute myeloid leukaemia (AML) [7, 8]. The CD66a, b antigens are members of carcinoembryonic antigen (CEA) family, which are expressed in granulocytes and B cells [9]. In a few clinical trials, ^90^Y- besilesomab also known as ^90^Y-anti-CD66 has successfully been used for conditioning regimens [10, 11]. Nevertheless, dosimetry is essential to confirm suitability for this treatment by evaluating the treatment dose received by the bone marrow, while safeguarding non-target organs, such as the liver, from unintended radiation exposure. Some studies have shown that a dose threshold of more than 40 Gy in bone marrow can cause permanent aplasia, whereas a dose threshold of more than 30 Gy in the liver was deemed to induce radiation-induced liver disease (RILD) [12, 13, 14]. A small proportion of clinical studies have demonstrated that anti-CD66 labelled with radioactive isotopes can efficiently deliver radiation to the bone marrow while keeping systemic toxicity low [15, 16, 17, 18]. Due to large inter-patient variability in the biokinetics and, consequently, in the toxicity, Kotzerke et al. and Kletting et al. [19, 20] emphasised the need for individual patient dosimetry in anti-CD66 RIT for accurate prediction of the therapeutic biodistributions. A further effort was made to simplify and establish dosimetry for anti-CD66 RIT in routine practice [21–23].

The need for multidisciplinary engagement to establish dosimetry-based personalised treatment in radionuclide therapy (RNT) has previously been emphasised [24]. Treatment must be optimised according to the patient’s dosimetry to provide personalised medicine. If patient-individualised absorbed doses are calculated in RNT, special attention must be paid to accurate data collection, quantification, analysis, and measurement of individual organ volumes. To further improve reliability and inform on the accuracy of treatment prediction, uncertainty analysis should be incorporated into the dose calculations. Most studies have relied on phantom measurements or simulation studies to measure the accuracy of absorbed dose calculations in RNT [27, 28, 29]. However, because of the diversity of dosimetry data, phantom experiments or simulations are not always sufficient to validate the accuracy of dose measurements for the patient population. Therefore, every patient should be evaluated individually to assess the accuracy of the results [26]. The accuracy of these measurements can easily be described by uncertainty. Many sources of uncertainties are associated with internal dosimetry used in radionuclide therapy [25, 26].

The most widely accepted method of internal dose calculation is the medical internal radiation dose (MIRD) schema [30]. Most studies on absorbed dose measurements have focused on only one or a few aspects of the MIRD schema specific to system calibration and activity quantification for uncertainty measurement [31, 32]. However, Internal dosimetry is a multi-step process (including activity measurement, system calibration, volume delineation, time activity curve analysis, and dose analysis), and each step propagates specific uncertainty in the final absorbed dose [29]. Therefore, the calculation of total absorbed dose uncertainty should include each step. A few aspects of uncertainty within the dosimetry steps have already been addressed [33, 34]. Besides, Finocchiaro et al. [26] performed the uncertainty analysis of tumour absorbed dose by incorporating specific uncertainty associated with different steps of dosimetry.

The European Association of Nuclear Medicine (EANM) published guidelines on uncertainty estimation for absorbed dose calculations in RNT, which applies the law of propagation of uncertainty (LPU) and can be implemented in clinical practice [25]. In a few single-time point (STP) dosimetry studies, the accuracy and difference of measurements have been tested using direct comparison with standard multi-time point (MTP) dosimetry [35, 36, 37]. However, the difference between standard MTP measurements and STP results is a measure of potential error, not uncertainty [36, 38], and no published data to date has shown a result of head-to-head uncertainty comparison between these two approaches and addressed uncertainty analysis in each step of STP dosimetry. Our primary aim was to calculate the uncertainties associated with different variables of 24-hour STP absorbed dose calculations and compare the results with MTP in the bone marrow and liver. This study also determines the parameters that greatly influence the dose calculations in STP dosimetry and assesses its appropriateness when applied to dosimetry guided bone marrow conditioning with ^90^Y besilesomab RIT.

**Fig. 1:**
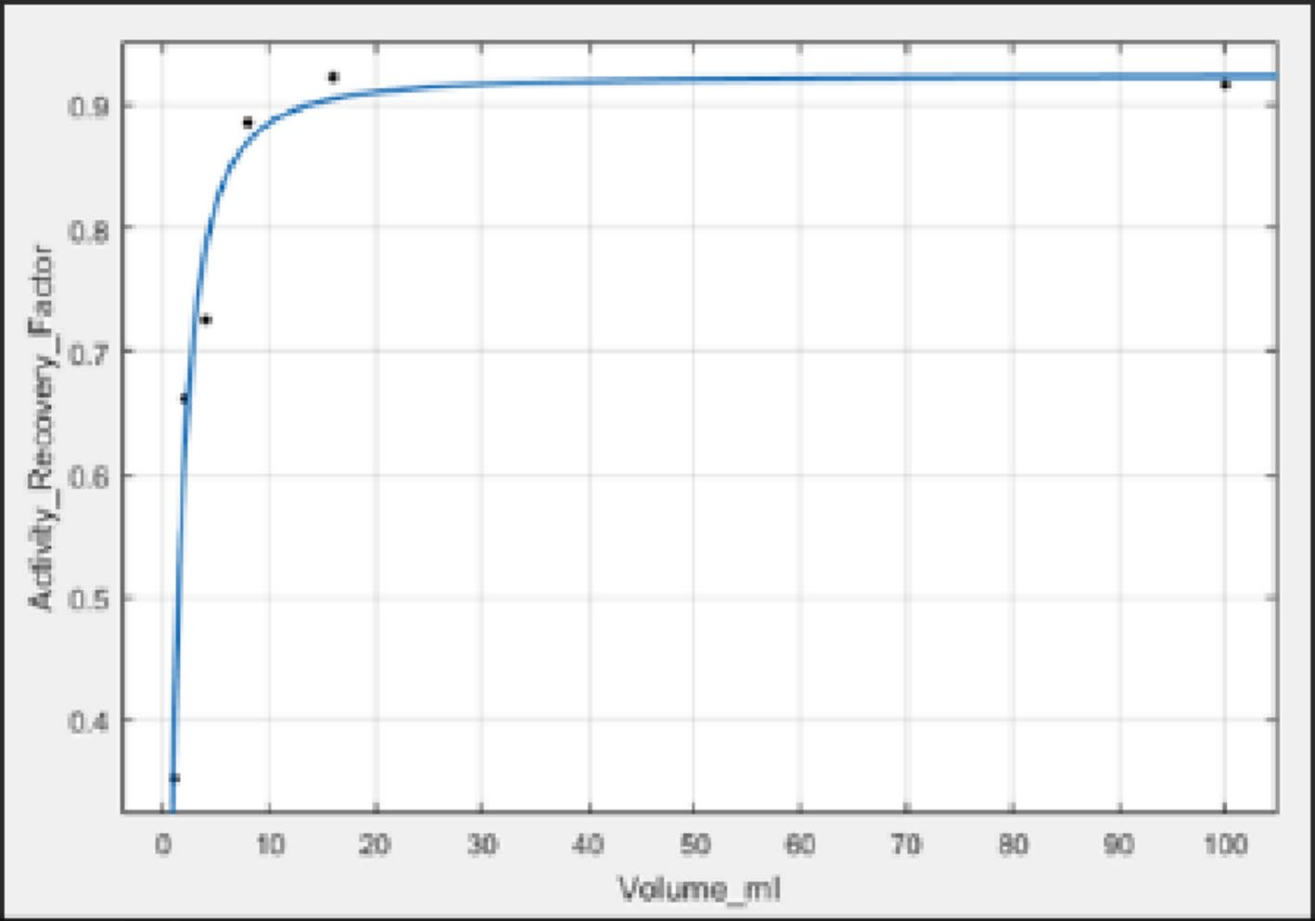
A curve between volume and recovery coefficient for the correction of partial volume effects

## Materials and methods

### Patients

In this retrospective study, 28 patients participated in three separate randomised clinical trials between 2013 and 2017 (NCT01521611, ISRCTN11933044 and ISRCTN13400668). This study was approved by the UK National Research Ethics Committee (RECs), the Administration of Radioactive Substances Advisory Committee (ARSAC) and the Medicines and Healthcare Products and Regulatory Authority (MHRA). Patients were recruited for haematopoietic stem cell transplant (HSCT) as a treatment for myeloma, acute myeloid leukaemia (AML), or chronic myeloid leukaemia (CML). Each patient received an intravenous injection of ^111^In besilesomab (0.17 ± 0.01GBq) for pre-therapeutic dosimetry and was subsequently treated with ^90^Y besilesomab (2.43 ± 0.53GBq). A dosimetric analysis was performed on bone marrow and liver for each patient.

**Table 1:** SPECT/CT acquisition parameters

SPECT/CT Acquisition parameters	
Energy windows	EM1: 245 keV ± 10%, EM2: 171 keV ± 10%, SC1: 209 keV ± 5%
Mode	H
Arc/Detector	180 degrees (Head 1 and 2 combined)
Projections	120 (3-degree step, 60 views per head), Step and Shoot, clockwise
Time per view	20 s
Matrix	256 × 256
Zoom	1.0
Pan	0
Body contour	ON
CT	Automated exposure control Tube voltage: 130 kV mAs: 30 Pitch: 1.0 Slice thickness: 5 mm 512 × 512 matrix Rotation time 0.6s Reconstruction: FBP

### Image acquisition

All acquisitions were performed post administration of ^111^In-besilesomab using General Electric Infinia Hawkeye SPECT-CT scanner (GE Healthcare, USA). A medium energy general purpose collimator (MEGP) was used for imaging. All data were acquired with the patient in the supine position. The imaging regimen comprised three sequential SPECT/CT scans of the abdomen, typically at 24, 72 and 96 h post-injection. Due to scheduling, scanner availability and patient variability, the actual acquisition times ranged from 19.7 to 46.9 h for the first scan, 54 to 72.5 h for the second scan, and 73.7 to 98.7 h for the third scan. Patients were encouraged to empty their bladder before each scan. The full acquisition parameters are detailed in Table 1. Two photopeaks were used, with an intermediate scatter window to estimate scatter while maintaining adequate counting statistics for quantitative imaging. Acquired data were reconstructed using a 3D ordered-subset expectation maximisation (OSEM) algorithm (4 iterations; 8 subsets; Butterworth filter (critical frequency 0.4 cm and power 10). Resolution recovery was not available on the imaging system. The reconstructed voxel size of the image volume was 4.4 mm^3^. All acquisition and reconstruction parameters were chosen as per the trial’s requirements.

**Fig. 2:**
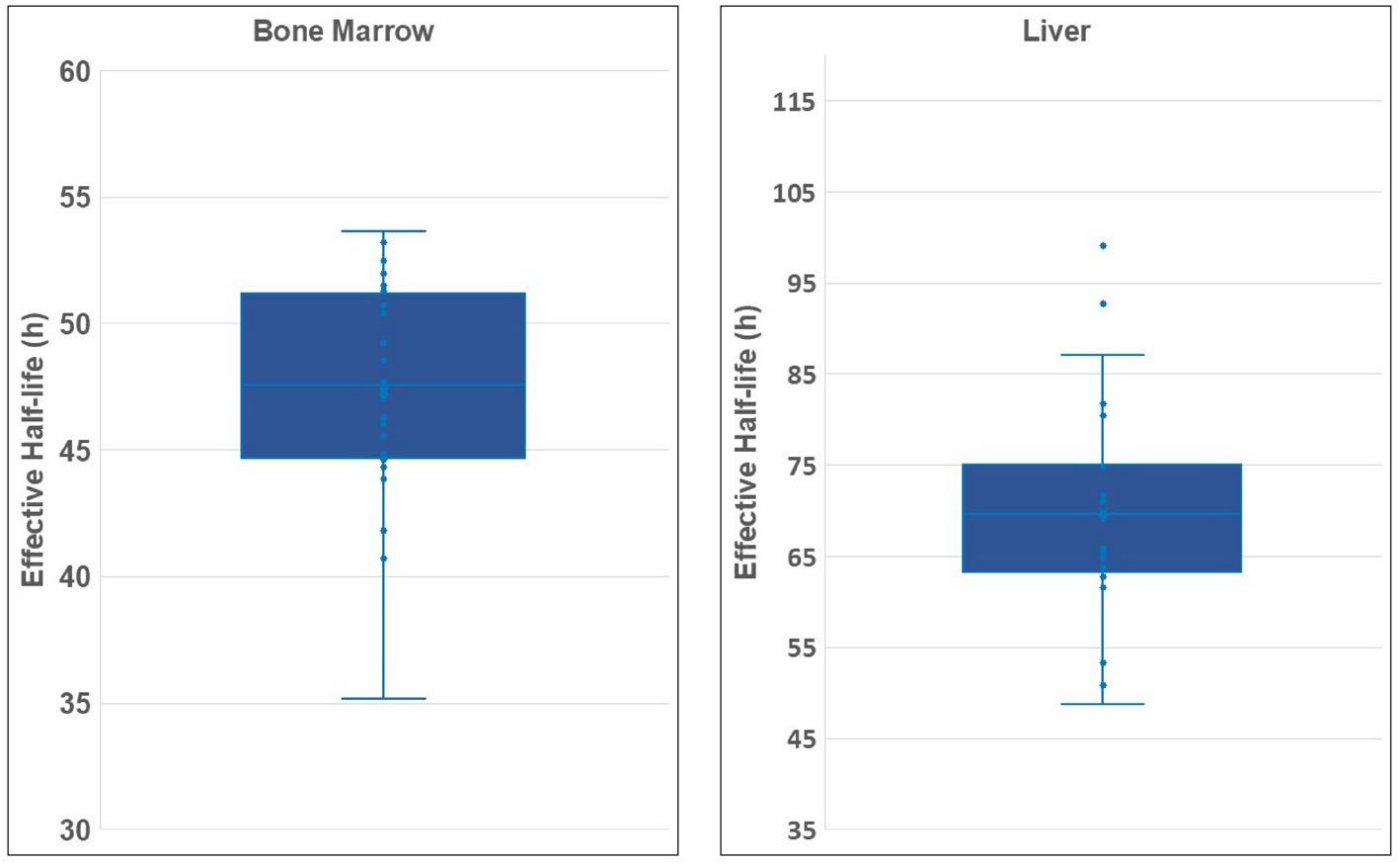
Box chart shows the distribution of effective half-life of ^90^Y-anti-CD66 in the bone marrow and liver calculated based on MTP

### Dosimetry procedure for MTP

^111^In-besilesomab SPECT/CT images were used to perform patient-specific dosimetry before the treatment with ^90^Y-besilesomab RIT. All acquired images were corrected for attenuation and scatter to maintain quantitative accuracy. Scatter in the upper photopeak was estimated using a dual-energy window approach based on counts measured in the scatter window. The scatter contribution in the lower photopeak was estimated by linear extrapolation between the top edge of the upper photopeak and the centre of the scatter window [39]. A Jaszczak phantom with fillable spheres (113 ml, 16 ml, 8 ml, 4 ml and 2 ml) was used to determine recovery coefficients (R) to further correct for PVE (Fig. 1). The following function was fitted to the phantom data and used to determine volume specific correction factors:

**Table 2:** Patient characteristics

Patient number	Age at transplant yrs	Sex	Diagnosis	Type of transplant
01	45	M	Myeloma	Autologous (1st)
02	55	M	Myeloma	Autologous (1st)
03	58	M	Myeloma	Autologous (1st)
04	65	F	Myeloma	Autologous (1st)
05	55	F	Myeloma	Autologous (1st)
06	48	M	Myeloma	Autologous (2nd)
07	58	M	AML M6	Allogeneic (sib)
08	20	F	AML M5a t(9;11)	Allogeneic (sib)
09	43	M	Myeloma	Autologous (1st)
10	59	F	Myeloma	Autologous (1st)
11	66	M	Myeloma	Autologous (1st)
12	56	M	Myeloma	Allogeneic (sib)
13	56	M	Myeloma	Allogeneic (sib)
14	53	F	Myeloma	Autologous (1st)
15	62	F	Myeloma	Autologous (1st)
16	65	M	Myeloma	Autologous (1st)
17	65	M	Myeloma	Autologous (1st)
18	68	F	Myeloma	Autologous (1st)
19	60	M	Myeloma	Autologous (1st)
20	62	M	CML-CP	Allogeneic (sib)
21	61	M	CML-AP	Allogeneic (VUD)
22	61	M	AML M4, FLT3 mutation. Primary refractory.	Allogeneic (VUD)
23	54	M	Myeloma, Lambda LCD	Allogeneic (sib)
24	24	M	Secondary AML, (previous HD. Mediastinal radiotherapy, autoHSCT)	Allogeneic (VUD)
25	55	M	Secondary AML	Allogeneic (sib)
26	45	M	Relapsed Myeloma IgA kappa	Allogeneic (VUD)
27	36	F	AML M7	Allogeneic (sib)
28	57	M	Relapsed AML M4	Allogeneic (VUD)

1 $$ f\left( x \right) = 1 - (1/(1 + (x/a)^{b} $$

Where a and b are fitting coefficients, and x is the physical volume of the spherical insert. The calibration factor (CF) for ^111^In was determined using a cylindrical uniformity phantom without inserts filled with a homogenous ^111^In solution. The volume of interest (VOI) for CF calculation was defined using a 10% threshold. This factor was checked annually as part of the clinical trial. All phantom acquisitions were performed using the same parameters as used for patient imaging. The CF was used to convert the total counts within a defined VOI on the reconstructed SPECT images into activity (MBq). VOI segmentation was performed using manual delineation of the bone marrow and liver. The percentage injected activity (%ID) at each time point and the effective half-life for ^111^In-besilesomab were also estimated. Bone marrow volume was estimated based on scaling from L2-L4 vertebrae marrow volume which was assigned to 6.7% of total WB marrow volume [40]. Volume for the liver was measured using the low-dose CT data of the SPECT/CT acquisition.

**Fig. 3:**
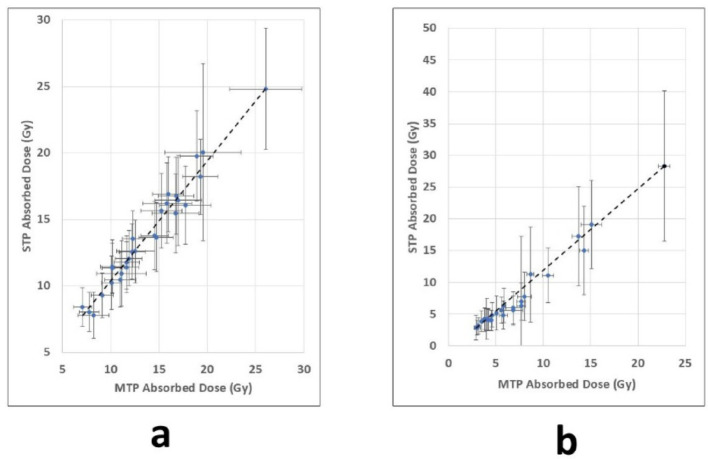
Scatter plot of MTP versus STP with horizontal and vertical error bars representing the calculated uncertainties in bone marrow **a** and liver **b** absorbed dose. A line of identity is included to illustrate the expected perfect agreement between measurements

The ^90^Ybesilesomab uptake within each organ. $\:{\boldsymbol{A}}_{\boldsymbol{t}}^{\boldsymbol{Y}90}$at each time point, $\:\boldsymbol{t}$ was then estimated using the measured ^111^In activity, $\:{\boldsymbol{A}}_{\boldsymbol{t}}^{\boldsymbol{I}\boldsymbol{n}111}$, corrected for the different physical half-lives and the ratio of administered activities of the two radionuclides according to the following expression:

2 $$ A_{t}^{{Y90}} = \frac{{A_{{inj}}^{{Y90}} }}{{A_{{inj}}^{{In111}} }}A_{t}^{{In111}} e^{{\left( {\lambda _{{Y90}} - \lambda _{{In111}} } \right).t}} $$

Where $\:{\boldsymbol{\lambda\:}}_{\boldsymbol{Y}90}$ and $\:{\boldsymbol{\lambda\:}}_{\boldsymbol{I}\boldsymbol{n}111}$ are the physical decay constants of ^90^Y and ^111^In respectively and $\:{\boldsymbol{A}}_{\boldsymbol{i}\boldsymbol{n}\boldsymbol{j}}^{\boldsymbol{Y}90}$ and $\:{\boldsymbol{A}}_{\boldsymbol{i}\boldsymbol{n}\boldsymbol{j}}^{\boldsymbol{I}\boldsymbol{n}111}$ are the injected activities.

For the multi time point approach a patient specific effective half-life for ^90^Y-besilesomab was estimated by fitting a single exponential function to the temporal activity data.

**Fig. 4:**
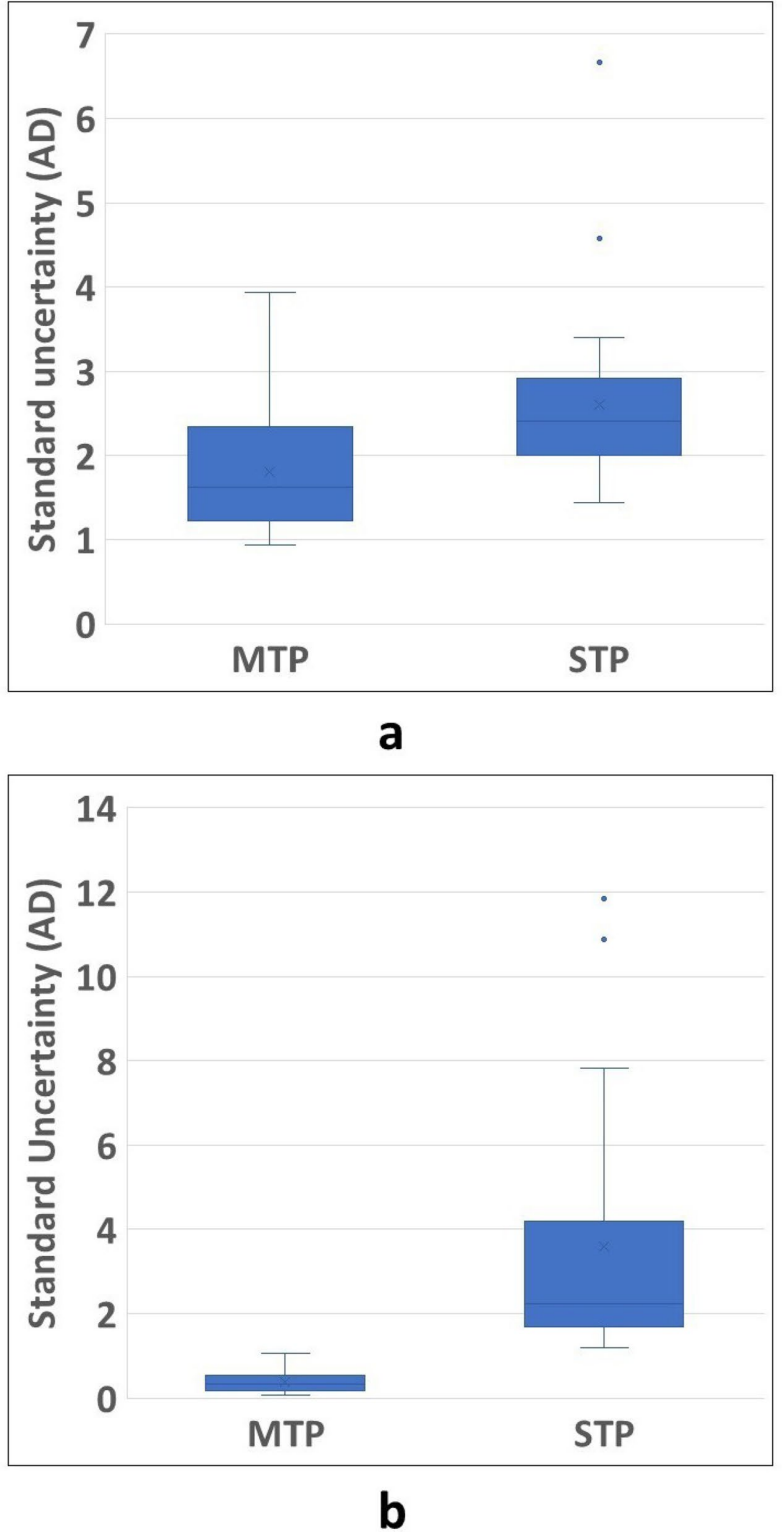
A comparison of the standard uncertainty of absorbed dose between MTP and STP for bone marrow **a** and liver **b**.

The time integrated activity ($\:\stackrel{\sim}{\boldsymbol{A}})$ was then calculated using

3 $$ \tilde{A} = ~\frac{{A_{0} }}{\lambda } $$

Where A_0_ is the ^90^Y activity at 0 h and λ is effective decay constant.

In the single time point study, dose data were calculated using only the 24 h time point. Mean population effective decay constants (λ_mean_) for both liver and bone marrow (Fig. 2) were determined from the complete patient datasets [41]. ^90^Y time integrated activity was then derived using:

4 $$ \tilde{A} = \frac{{A_{t} e^{{\lambda _{{mean}} .t}} }}{{\lambda _{{mean}} }} $$

Where $\:{\boldsymbol{A}}_{\boldsymbol{t}}$ is the ^90^Y activity measured at time t, nominally at t = 24 h.

For each approach the total absorbed dose to the organs was estimated by multiplying the calculated time integrated activity with a weight-scaled S value [42].

5 $$ Dose~\left( {Gy} \right) = ~\tilde{A} \times S_{{patient}} $$

Where $\:\stackrel{\sim}{\boldsymbol{A}}$ is the time integrated activity in the organ of interest in MBq.h and S_patient_ is S values in Gy/MBq-h.

### Uncertainty analysis in MTP

The uncertainty associated with each MTP parameter was calculated based on the EANM guidelines [25]. The pertinent expression from the guidelines is the uncertainty in the time integrated activity, which for conventional MTP dosimetry takes the form,

**Table 3:** Standard and fractional uncertainty associated with bone marrow and liver absorbed dose

	Bone marrow		Liver	
	MTP	STP	MTP	STP
Absorbed dose (Gy)	13.75 ± 4.31	13.77 ± 4.02	6.93 ± 4.60	7.41 ± 5.91
Standard uncertainty	1.81 ± 0.76	2.60 ± 1.03	0.38 ± 0.27	3.59 ± 2.88
Fractional uncertainty (%)	13.18 ± 3.46	18.75 ± 3.22	5.77 ± 3.13	49.78 ± 25.36

6 $$ \left[ {\frac{{u\left( {\tilde{A}} \right)}}{{\tilde{A}}}} \right]^{2} = \left[ {\frac{{u\left( {A_{0} } \right)}}{{A_{0} }}} \right]^{2} + \left[ {\frac{{u\left( \lambda \right)}}{\lambda }} \right]^{2} - 2\frac{{u\left( {A_{0} ,\lambda } \right)}}{{A_{0} \lambda }} + \left[ {\frac{{u\left( {A_{t} } \right)}}{{A_{t} }}} \right]^{2} $$

Where $\:u({A}_{0}$) and $\:u(\lambda\:$) are the standard uncertainty in the TAC fitting parameters $\:{A}_{0}$ and $\:\lambda\:,\:$ and $\:u({A}_{0},\lambda\:$) is the covariance between them. The uncertainty associated with activity, $\:u\left({A}_{t}\right)$, is a systematic uncertainty term with equal fractional uncertainty across all measurement points, provided by Eq. (7)

7 $$\:{\left[\frac{u\left({A}_{t}\right)}{{A}_{t}}\right]}^{2}={\left[\frac{u\left({R}_{v}\right)}{{R}_{v}}\right]}^{2}+{\left[\frac{u\left(C\right)}{C}\right]}^{2}-2\frac{u\left({R}_{v},C\right)}{{R}_{v}C}.$$

Where $\:\boldsymbol{u}({\boldsymbol{R}}_{\boldsymbol{v}}$) is the uncertainty associated with the applied recovery term and $\:\boldsymbol{u}(\boldsymbol{C}$) is the standard uncertainty in the measure count rate. $\:\boldsymbol{u}({\boldsymbol{R}}_{\boldsymbol{v}},\boldsymbol{C}$) is the covariance between these two parameters which are each dependent on a singular volume estimate. In this study as a conversion between ^111^In and ^90^Y activity was required an extension to this expression is required to account for the conversion between the two isotopes, which assuming negligible uncertainty in the physical half-lives can be expressed as,

8 $$ \left[ {\frac{{u\left( {A_{t}^{{Y90}} } \right)}}{{A_{t}^{{Y90}} }}} \right]^{2} = \left[ {\frac{{u\left( {A_{{inj}}^{{Y90}} } \right)}}{{A_{{inj}}^{{Y90}} }}} \right]^{2} + \left[ {\frac{{u\left( {A_{{inj}}^{{In111}} } \right)}}{{A_{{inj}}^{{In111}} }}} \right]^{2} + \left[ {\frac{{u\left( {A_{t}^{{In111}} } \right)}}{{A_{t}^{{In111}} }}} \right]^{2} $$

Where the uncertainty in the injected activities, and can be taken as the uncertainty in the dose calibrator measurements. For a field calibrator, the accuracy should be calibrated to within 5% of a primary reference standard [43]. Therefore, assuming an equal probability of the true activity to lie anywhere between − 5% and + 5%, i.e. a uniform distribution of possible values, using expression 7 of the GUM [44] the relative uncertainty associated with a calibrator activity measurement is,

**Fig. 5:**
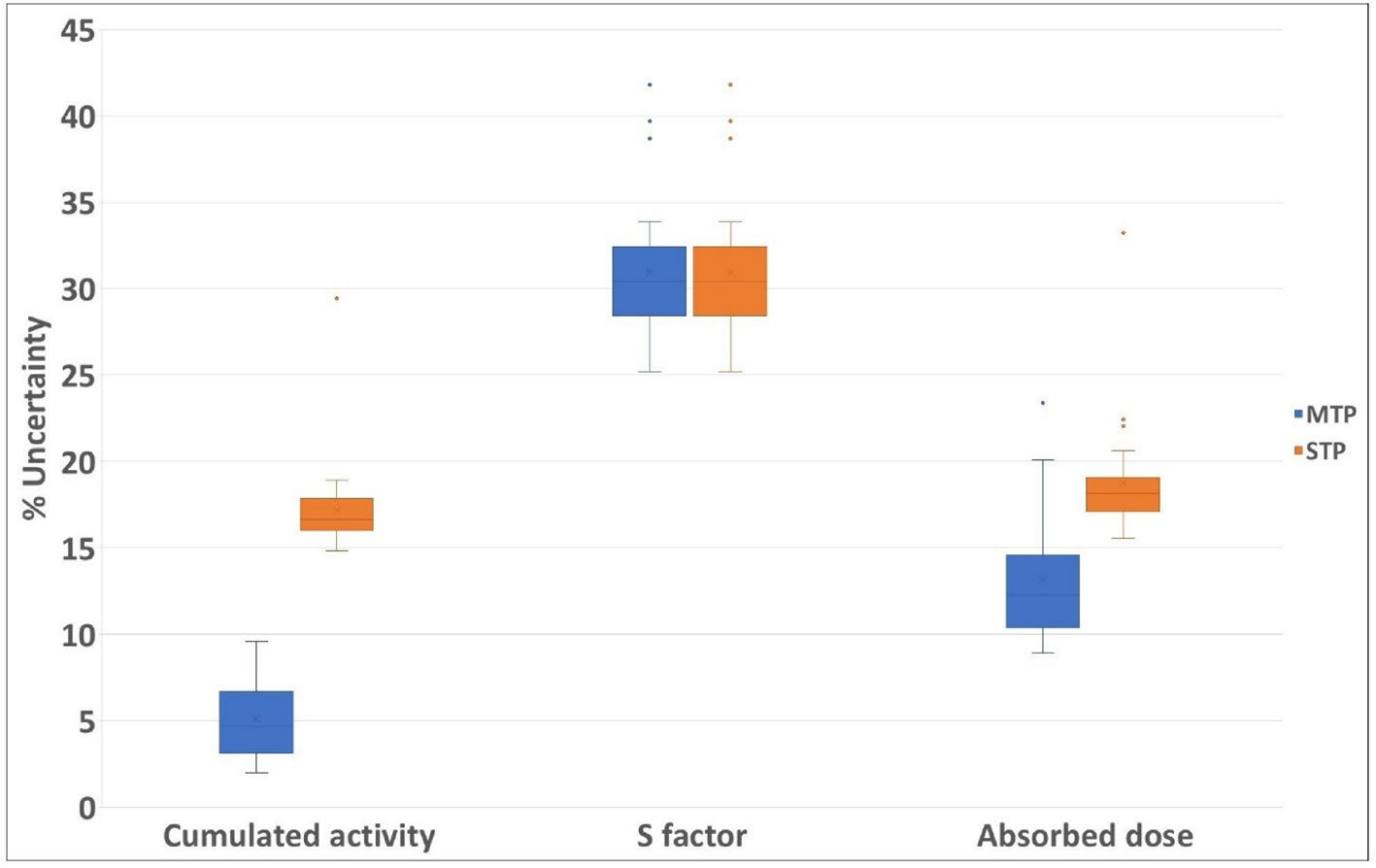
A comparison of fractional percentage uncertainty of key dosimetric parameters between MTP and STP for bone marrow

9 $$ \left[ {\frac{{u\left( {A_{{inj}}^{{Y90}} } \right)}}{{A_{{inj}}^{{Y90}} }}} \right]^{2} = \left[ {\frac{{u\left( {A_{{inj}}^{{In111}} } \right)}}{{A_{{inj}}^{{In111}} }}} \right]^{2} = \frac{{a^{2} }}{3} = \frac{{\left( {0.05} \right)^{2} }}{3} = 2.9\% , $$

Where $\:\boldsymbol{a}$ is the acceptable limit of accuracy of the field calibrator. Combining expressions 7 and 9 then gives

10 $$ \left[ {\frac{{u\left( {A_{t} } \right)}}{{A_{t} }}} \right]^{2} = 0.029 + 0.029 + \left[ {\frac{{u\left( {R_{v} } \right)}}{{R_{v} }}} \right]^{2} + \left[ {\frac{{u\left( C \right)}}{C}} \right]^{2} - 2\frac{{u\left( {R_{v} ,C} \right)}}{{R_{v} C}} $$

and

11 $$ \left[ {\frac{{u\left( {\tilde{A}} \right)}}{{\tilde{A}}}} \right]^{2} = \left[ {\frac{{u\left( {A_{0} } \right)}}{{A_{0} }}} \right]^{2} + \left[ {\frac{{u\left( \lambda \right)}}{\lambda }} \right]^{2} - 2\frac{{u\left( {A_{0} ,\lambda } \right)}}{{A_{0} \lambda }} + \left[ {\frac{{u\left( {R_{v} } \right)}}{{R_{v} }}} \right]^{2} + \left[ {\frac{{u\left( C \right)}}{C}} \right]^{2} - 2\frac{{u\left( {R_{v} ,C} \right)}}{{R_{v} C}} + 0.058 $$

### Uncertainty analysis in STP

In the STP dosimetry method, time integrated activity was estimated by assuming that the patient has an effective half-life equal to that of the population mean, provided in expression 4.

**Fig. 6:**
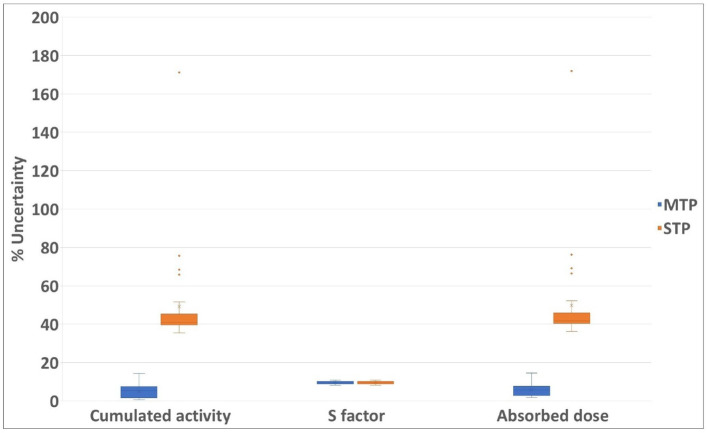
A comparison of fractional percentage uncertainty of key dosimetric parameters between MTP and STP for the liver

The uncertainty $\:u\left(\stackrel{\sim}{A}\right)$, is a combination of uncertainty in all input and can therefore be written.

12 $$ u^{2} \left( {\tilde{A}} \right) = ~\left( {\frac{{\partial \tilde{A}}}{{\partial A}}} \right)^{2} u^{2} \left( {A_{t} } \right) + \left( {\frac{{\partial \tilde{A}}}{{\partial \lambda }}} \right)^{2} u^{2} \left( \lambda \right) + \left( {\frac{{\partial \tilde{A}}}{{\partial t}}} \right)^{2} u^{2} \left( t \right) $$

Assuming the uncertainty in $\:t\:$is negligible, Eq. (12) will be

13 $$ u^{2} \left( {\tilde{A}} \right) = ~\left( {\frac{{\partial \tilde{A}}}{{\partial A}}} \right)^{2} u^{2} \left( A \right) + \left( {\frac{{\partial \tilde{A}}}{{\partial \lambda }}} \right)^{2} u^{2} \left( \lambda \right) $$

Solving partial derivatives

14 $$ u^{2} \left( {\tilde{A}} \right) = \left[ {\frac{{e^{{\lambda .t}} }}{\lambda }A_{t} \left( {\frac{{\lambda .t.e^{{\lambda .t}} - e^{{\lambda .t}} }}{{\lambda ^{2} }}} \right)} \right]^{2} u^{2} \left( {A_{t} } \right) + \left[ {\frac{{\lambda .t.A_{t} e^{{\lambda .t}} - A_{t} e^{{\lambda .t}} }}{{\lambda ^{2} }}} \right]^{2} u^{2} \left( \lambda \right) $$

Which after substituting for $\:\stackrel{\sim}{A}$, gives

15 $$ u^{2} \left( {\tilde{A}} \right) = ~\left( {\frac{{\tilde{A}}}{{A_{t} }}} \right)^{2} u^{2} \left( {A_{t} } \right) + \left( {\tilde{A}.t - \frac{{\tilde{A}}}{\lambda }} \right)^{2} u^{2} \left( \lambda \right) $$

Which can be re-expressed as

16 $$ \left[ {\frac{{u\left( {\tilde{A}} \right)}}{{\tilde{A}}}} \right]^{2} = \left[ {\frac{{u\left( {A_{t} } \right)}}{{A_{t} }}} \right]^{2} + \left[ {u\left( \lambda \right).t - \frac{{u\left( \lambda \right)}}{\lambda }} \right]^{2} $$

Finally, as with the case of MTP dosimetry, the uncertainty associated with the activity measurement and ^111^In and ^90^Y conversion is included to give

17 $$ \left[ {\frac{{u\left( {\tilde{A}} \right)}}{{\tilde{A}}}} \right]^{2} = \left[ {u\left( \lambda \right).t - \frac{{u\left( \lambda \right)}}{\lambda }} \right]^{2} + \left[ {\frac{{u\left( {R_{v} } \right)}}{{R_{v} }}} \right]^{2} + \left[ {\frac{{u\left( C \right)}}{C}} \right]^{2} - 2\frac{{u\left( {R_{v} ,C} \right)}}{{R_{v} C}} + 0.058 $$

### Statistical analysis

All statistical analyses were performed using R statistical software (4.3.2). All the calculations of uncertainty analysis associated with each parameter of absorbed dose were calculated using Excel 2010. Box plots were used to visualise the uncertainties associated with key parameters of the dosimetry calculation in MTP and STP dosimetry. Total standard uncertainties associated in MTP and STP absorbed dose were also represented using box plots. The relationship between fractional AD uncertainty and dosimetry parameters was assessed using regression analysis. Statistical differences between MTP and STP uncertainty measurements were analysed using the Wilcoxon signed-rank test, as the data were not normally distributed.

**Table 4:** Standard and fractional uncertainty associated with each parameter of MTP dosimetry in the bone marrow and liver

	Bone marrow		Liver	
	Standard uncertainty	Fractional uncertainty (%)	Standard uncertainty	Fractional uncertainty (%)
Calibration factor	0.43	5	0.43	5
^111^In to ^90^Y conversation	0.84 ± 0.002	1.73 ± 0.18	2.31 ± 0.02	3.24 ± 0.59
Volume	18.4 ± 4.06	31.3 ± 3.92	181.58 ± 27.30	9.83 ± 0.73
Recovery coefficient	0.01 ± 0.004	1.38 ± 0.005	0.017 ± 0.002	1.78 ± 0.002
Count rate	186,536 ± 42,544	14.28 ± 1.65	247,390 ± 37,839	4.75 ± 0.34
Curve fitting	31.51 ± 23.11	7.12 ± 4.58	0.008 ± 0.009	8.32 ± 5.77
Time integrated activity	2950 ± 1750	5.12 ± 2.20	1265 ± 1060	5.13 ± 3.49
S-factor	0.000025 ± 0.000015	30.93 ± 3.88	0.0000077 ± 0.000002	9.63 ± 0.71
Absorbed dose	1.81 ± 0.76	13.18 ± 3.46	0.38 ± 0.27	5.77 ± 3.13

## Result

### Patient characteristics

Twenty-eight patients (mean age 54.4 years, range 20–68, 8 females, 20 males) with history of multiple myeloma, AML or CML were included into the study. The detailed patient characteristics are summarised in Table 2.

**Fig. 7:**
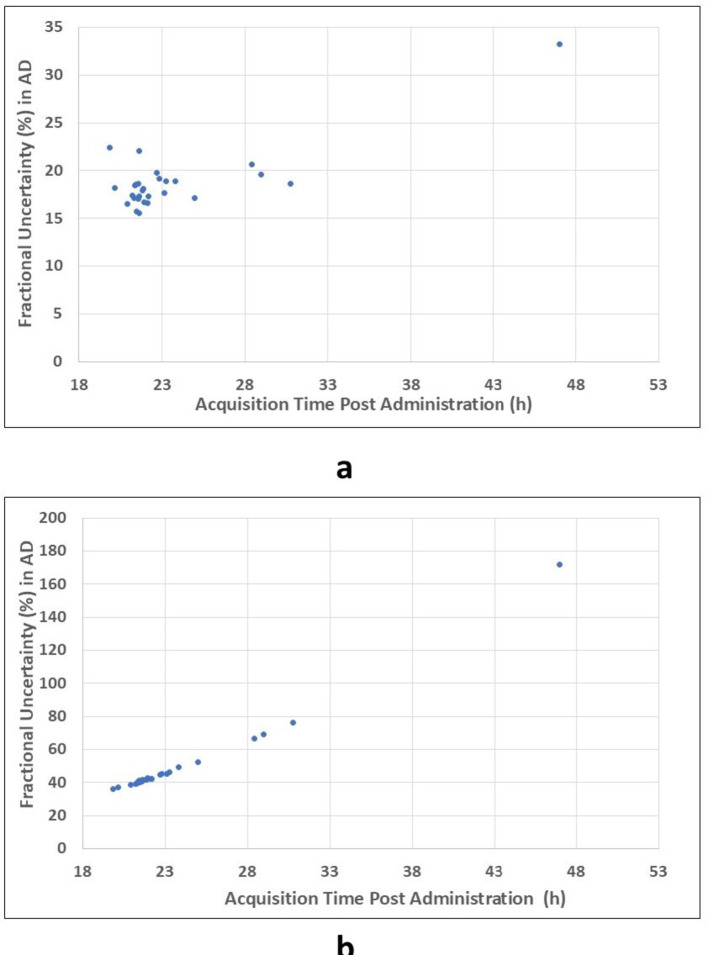
Correlation between absorbed dose uncertainty (y axis) and scan time post administration (x axis) in bone marrow **a** and liver **b**.

### Organ dose results

The mean absorbed dose per unit administered activity (DpA) to BM was 5.8 ± 1.7 mGy/MBq (Range: 2.93–9.25) with MTP and 5.8 ± 1.6 mGy/MBq (Range: 3.30–9.38) with STP, and to the liver was 2.9 ± 1.9 mGy/MBq (Range: 1.26–8.70) with MTP and 3.1 ± 2.4 mGy/MBq (Range: 1.34–11.02) with STP. The median DpA determined by STP was compared with MTP and found to increase by 0.54% in BM and decrease by 13.7% in the liver.

**Fig. 8:**
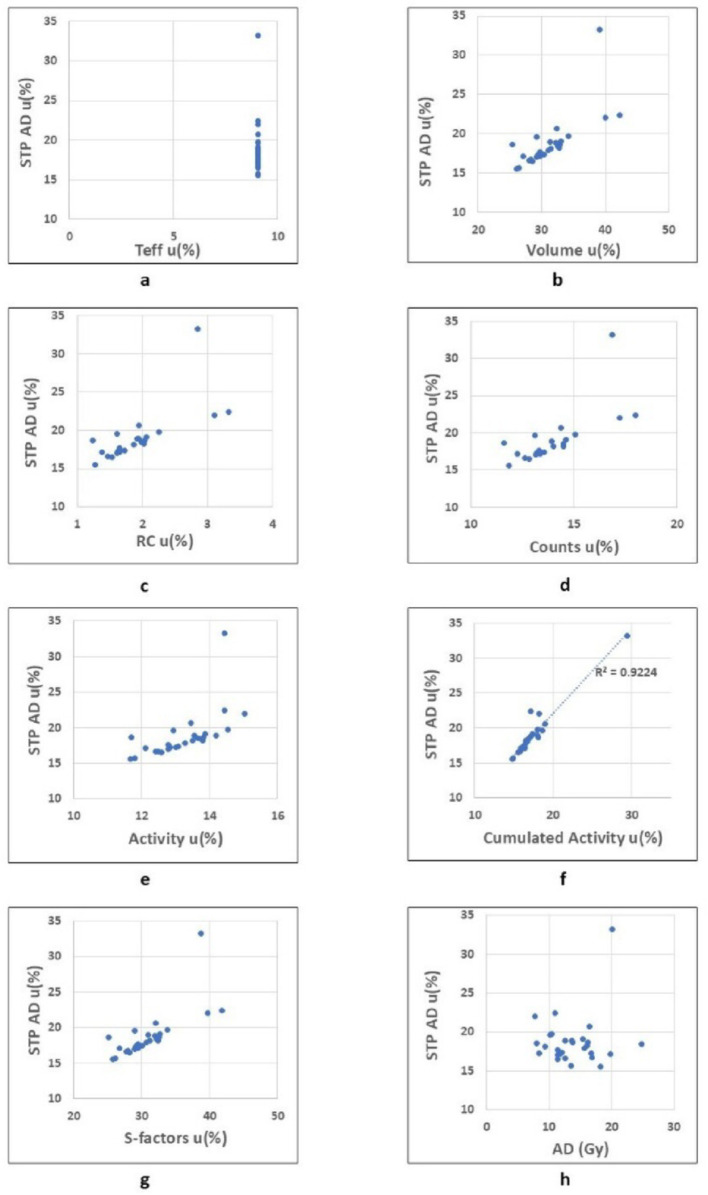
Correlation between STP absorbed dose uncertainty and Teff **a**, volume **b**, RC **c**, counts **d**, activity **e**, time integrated activity **f** and S-factors uncertainties **g** in bone marrow. The graph h shows the correlation between STP absorbed dose (Gy) and absorbed dose uncertainty

### Uncertainty analysis results

Using the full temporal dataset, mean uncertainty estimates were 13.2% with a range of 8.9% to 23.4% and 5.8% with a range of 1.9 to 14.5% to the bone marrow and liver, respectively. These uncertainties increased to 18.8 (15.5–33.2%) and 49.8% (36.3-171.9%) when using the single 24-hour time point for dosimetry. The absorbed dose uncertainty in STP was significantly higher compared to the MTP for the bone marrow and liver (*p* < 0.001).

**Table 5:** Standard and fractional uncertainty associated with each parameter of STP dosimetry in the bone marrow and liver

	Bone marrow		Liver	
	Standard uncertainty	Fractional uncertainty (%)	Standard uncertainty	Fractional uncertainty (%)
Population Teff	0.81	1.73 ± 0.2	2.18	3 ± 0.5
Volume	18.4 ± 4.06	31.3 ± 3.92	181.58 ± 27.30	9.83 ± 0.73
Recovery coefficient	0.01 ± 0.004	1.38 ± 0.005	0.017 ± 0.002	1.78 ± 0.002
Count rate	186,536 ± 42,544	14.28 ± 1.65	247,390 ± 37,839	4.75 ± 0.34
Activity	76.14 ± 31.12	13.22 ± 0.86	11.15 ± 9.21	5.19 ± 0.33
Time integrated activity	9581 ± 4038	17.21 ± 2.56	13,127 ± 10,938	49.10 ± 25.34
S-factor	0.000025 ± 0.000015	30.93 ± 3.88	0.0000077 ± 0.000002	9.63 ± 0.71
Absorbed dose	2.60 ± 1.03	18.75 ± 3.22	3.59 ± 2.88	49.78 ± 25.36

A scatter plot of MTP versus STP absorbed dose was generated with horizontal and vertical error bars representing the calculated uncertainties for each measurement, and a line of identity was included to visually assess the agreement between the two methods in the BM and liver (Fig. 3).

**Fig. 9:**
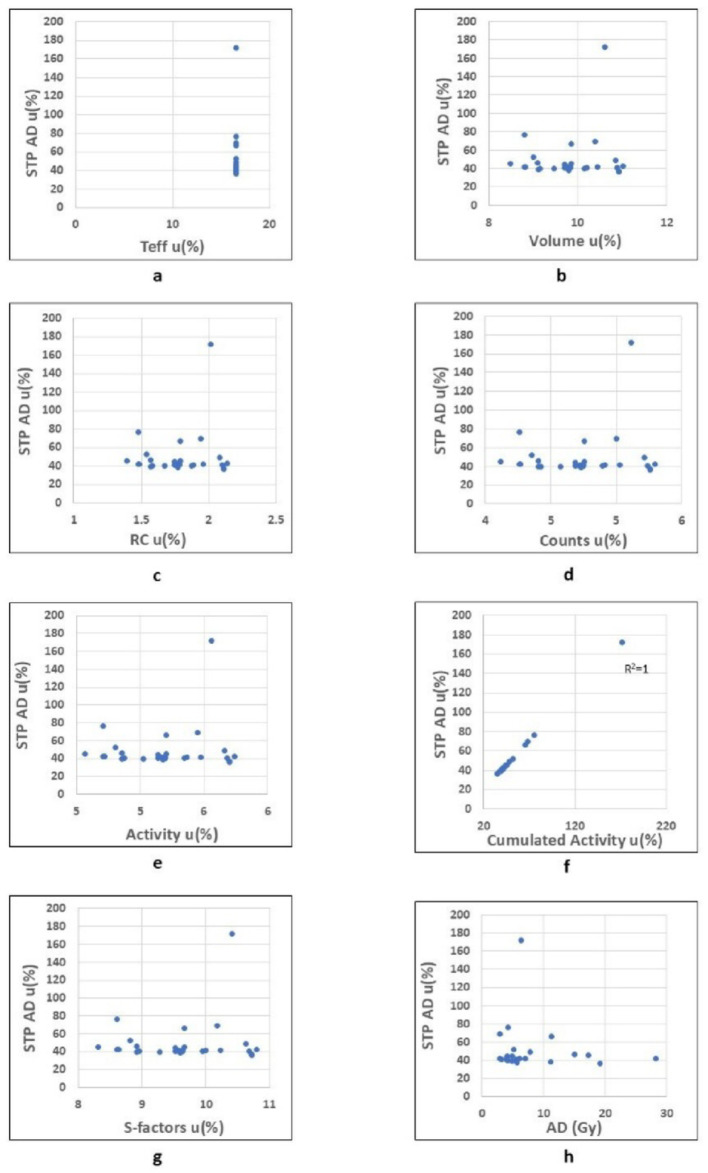
Correlation between STP absorbed dose uncertainty and Teff **a**, volume **b**, RC **c**, counts **d**, activity **e**, time-integrated activity **f** and S-factors uncertainties **g** in liver. The graph h shows the correlation between STP absorbed dose (Gy) and absorbed dose uncertainty

The median uncertainty determined by STP was compared with MTP and found to increase by half-fold in BM and increase by six-fold in the liver. The details of standard uncertainty associated with absorbed dose are mentioned in Table 3 and shown in Fig. 4. Tables 4 and 5 present the standard and fractional uncertainty associated with each dosimetry parameter. Figures 5 and 6 show the distribution of fractional uncertainty for key dosimetry parameters calculated using the MTP and STP methods in the BM and liver. A moderate positive relationship (R^2^ = 0.7) was noted between post-injection acquisition time and AD uncertainty with STP for BM, whereas a strong positive relationship (R^2^ = 1) was noted for the liver (Fig. 7). A poor correlation was noted between uncertainty associated with parameters used in the STP dosimetry chain (Teff, volume, RC, counts, activity, and S-factors) and AD uncertainty for BM and liver (Figs. 8 and 9). A positive relationship was noted between fractional time integrated activity and AD uncertainty with the STP method for BM (R^2^ = 0.93) and liver (R^2^ = 1). No association was noted between the calculated absorbed dose and fractional uncertainty in AD for BM and liver estimated using the STP method.

## Discussion

The EANM guidelines provide a schema of uncertainty analysis for each parameter along with MTP dosimetry workflows. For several steps in the dosimetry process, this schema was developed based on recommendations within the Guide to the Expression of Uncertainty in Measurement (GUM) [45]. Dosimetry is not yet widely adopted in RNT, while the uncertainty of absorbed dose calculations is seldom characterised. In this work, we used the EANM guidelines to estimate the uncertainty of critical organ dosimetry using MTP in RIT. We also provided a schema of uncertainty propagation and evaluated uncertainty in STP dosimetry calculation and compared the results with MTP. Compared with the existing scientific background, this study conducted a first-ever uncertainty analysis of the entire process of STP dosimetry calculation on patients treated with RIT. By establishing a benchmark for the typical level of uncertainty involved, our results offer valuable context to support users in interpreting dosimetry estimates to better inform clinical decisions.

In the MTP dosimetry, the volume delineation, S-factor, counts and curve fitting parameters are associated with the highest uncertainty value compared to other parameters. These results emphasised that accuracy in absorbed dose results is mainly influenced by the accuracy in VOI delineation, the number of counts acquired in the image and the time activity fitting used to calculate the time-integrated activity. The fractional uncertainty related to volume and S-factor in the liver was found to agree with the results of uncertainty analysis reported by Gear et al. [25]. The S-factor uncertainty is not a primary source of uncertainty as it mainly originates from the volume uncertainty [26]. We have found that the smaller bone marrow volume (L2-L5) has a significant impact on the greater uncertainty compared to the liver volume. Similar to our results, Gear et al. [25] also observed the significant impact of a smaller volume on uncertainty. The limited spatial resolution and voxelization of SPECT images are the key factors leading to the volume delineation uncertainty. However, these segmentation uncertainties can be reduced by using high-resolution images. The accuracy of organ segmentation can be improved by new-generation SPECT/CT scanners, which use advanced reconstruction algorithms incorporating point spread function or resolution modelling technology or by using diagnostic high-resolution computed tomography (CT) images or magnetic resonance imaging (MRI) to delineate a more precise outline [46–48]. A robust and consistent artificial intelligence (AI) based segmentation tool may also minimize operator-related volume-based uncertainties. The results of our study also show that fractional uncertainty associated with absorbed dose in BM and liver is lower than that of volume and S-factors uncertainty because of covariance effects within the dosimetry series. These findings are in line with studies that also addressed the effects of covariance within the dosimetry chain [25, 26]. Count-related uncertainties could be improved by using high-sensitivity SPECT. The fractional uncertainties in the fitting parameters for TAC in the liver are higher than that in BM, mainly due to the random error affecting fitting parameters, which begin to dominate as volume increases. However, it has been demonstrated that uncertainty in the absorbed dose rate is not influenced by random components of the fitting parameters [26]. It is known that uncertainties in TAC fitting are mainly affected by scan times, the number of time points and the fitting model employed [48]. These uncertainties may be reduced by the model averaging technique [49]. In our dataset, mono-exponential fitting adequately described the measured time–activity curves over the imaging interval (24–96 h), with high goodness-of-fit values (mean R² > 0.99 for bone marrow and liver). Residual analysis did not reveal systematic deviations from mono-exponential behaviour, which confirms the appropriateness of this model for the available data.

Our study results show that the fractional uncertainties associated with STP dosimetry are significantly higher than those associated with MTP. Volume and time-integrated activity are the parameters associated with maximum uncertainty. It is worth noting that uncertainties propagating to time-integrated activity in STP dosimetry are dominated by accuracy in the acquisition time and population-based half-life. Moreover, some uncertainties are also added by the assumption of single exponential excretion. The EANM dosimetry committee has also demonstrated the impact of different STPs in the time-integrated activity estimation [48]. The ^90^Y-besilesomab BM and liver effective half-life were in a range of 35–54 h and 49–99 h, respectively. The effective half-life tends to be overestimated when early imaging time points are missing, as the initial rapid clearance phase is not captured. The uncertainties propagating in the time-integrated activity were significantly higher in the liver than in the BM, which is due to higher uncertainty in the population half-life and selection of acquisition time for liver dosimetry. In a separate dosimetry study, we observed that the BM dose result in STP was in best agreement with MTP results at 24 h [50]. In contrast, in the case of liver dosimetry, a late time point scan such as 96 h p.i. improved agreement with MTP because the late portion of the time-activity curve contributes substantially to the absorbed dose estimate. However, we used a 24-hour time point for organ dosimetry with a slight loss of accuracy in liver dose results, as it is feasible in terms of patient comfort (short scan duration) and allows treatment planning on the day after ^111^In administration, which could be possible in one hospital visit with an overnight stay. The larger increase in uncertainty observed for liver dosimetry with the STP method is therefore partly related to the choice of the 24-hour imaging time point, which was primarily optimised for bone marrow dosimetry. While this time point provides a practical balance between dosimetry accuracy and clinical feasibility, a systematic evaluation of STP uncertainty across different acquisition times may further optimise liver dosimetry accuracy and would be an interesting area for future investigation.

The major uncertainties associated with the STP method may be reduced by estimating effective half-life from a larger cohort of patients, selecting optimal SPECT/CT imaging time, or using the best-fit function for calculating time integrated activity in STP dosimetry. Willowson et al. [51] used MTP based patient specific half time for first cycle and used this kinetics for STP dosimetry in subsequent cycles with aim to minimise the error associated with mean population half-life. A nonlinear mixed-effects (NLME) model may be used to determine best fit function for time integrated activity in STP dosimetry [52]. Assuming a maximum absorbed dose uncertainty of 25% as set by Peters et al. [53] in their study for tumours and organs, the results for total BM uncertainty in our patients were found to be lower than this reference limit. A good association was noted between absorbed dose uncertainty and acquisition time post-injection, indicating that the scan time is the primary factor affecting absorbed dose calculation accuracy in the STP method.

Although this study was conducted in patients undergoing radioimmunotherapy with ^90^Y-besilesomab, the findings may have broader relevance to other radionuclide therapies where STP dosimetry is frequently applied, such as ^177^Lu-DOTATATE and ^177^Lu-PSMA therapies [54, 55]. In these treatments, STP dosimetry has been explored as a practical alternative to MTP imaging to reduce patient burden and imaging resource use. However, pharmacokinetics and organ uptake characteristics differ between radiopharmaceuticals, which may influence the magnitude and propagation of uncertainties in absorbed dose calculations. Therefore, while the general principles identified in this study, such as the importance of acquisition timing, effective half-life estimation, and accurate time-integrated activity determination, are likely applicable to other radionuclide therapies, the magnitudes of uncertainty will vary depending on activity concentration in the organ, tracer kinetics and organ-specific clearance patterns. Further studies evaluating uncertainty propagation in STP dosimetry for therapies such as ^177^Lu-DOTATATE and ^177^Lu-PSMA would be useful.

Our study also had some limitations. We did not incorporate the uncertainties associated with image misregistration and operator-dependent volume estimation. The RC curve was generated based on the maximum concentration; however, the uncertainties with the RC might be higher with the mean activity concentration. The calculated absorbed dose was based on the MIRD schema, which is associated with some uncertainties, as this method does not consider tissue and activity heterogeneities. Our uncertainty results are based on a small sample size; more studies need to be performed with large patient cohorts to confirm the uncertainty results in RNT or RIT. We fitted the TAC curve with an assumption of a mono-exponential decay pattern; however, organs may follow a bi-exponential or tri-exponential decay pattern, for which uncertainties and results might vary. We did not check the effect of correction strategies on the result of uncertainty analysis as they were outside the scope of our study.

## Conclusion

This study is the first to analyse uncertainties related to MTP and STP organ-absorbed dose calculations in patients treated with RIT. The absorbed dose uncertainty in STP was significantly higher compared to the MTP method. It is strongly recommended to incorporate the uncertainty analysis for STP dosimetry parameters in routine clinical practice. The accuracy in the acquisition time, population-based half-life and fitting function for time activity curve is vital for minimising uncertainty and increasing the validity of STP dosimetry. Implementing uncertainty results would encourage clinicians to use STP dosimetry in treatment planning, which is less time consuming and easy to implement in clinical practice compared to MTP. In order to maximise the effectiveness of RIT or RNT, it is essential to improve the accuracy of absorbed dose calculations.

## Data Availability

The datasets analysed during the current study are available from the corresponding author on reasonable request.
